# Late-onset oro-facial dyskinesia in Spinocerebellar Ataxia type 2: a case report

**DOI:** 10.1186/s12883-020-01739-8

**Published:** 2020-04-27

**Authors:** Floriana Giardina, Giuseppe Lanza, Francesco Calì, Raffaele Ferri

**Affiliations:** 1 UO Neurologia, Ospedale Santa Maria del Carmine, APSS Trento. Corso Verona, 4, 38068 Rovereto, Italy; 2 Department of Surgery and Medical-Surgical Specialties, University of Catania, Via Santa Sofia, 78, 95123 Catania, Italy; 3 Oasi Research Institute-IRCCS, Via Conte Ruggero, 73, 94018 Troina, Italy

**Keywords:** Movement disorders, Oro-facial dyskinesia, Spinocerebellar ataxia

## Abstract

**Background:**

Genetic familiar causes of oro-facial dyskinesia are usually restricted to Huntington’s disease, whereas other causes are often missed or underestimated. Here, we report the case of late-onset oro-facial dyskinesia in an elderly patient with a genetic diagnosis of Spinocerebellar Ataxia type 2 (SCA2).

**Case presentation:**

A 75-year-old man complained of progressive balance difficulty since the age of 60 years, associated with involuntary movements of the mouth and tongue over the last 3 months. No exposure to anti-dopaminergic agents, other neuroleptics, antidepressants, or other drugs was reported. Family history was positive for SCA2 (brother and the son of the brother). At rest, involuntary movements of the mouth and tongue were noted; they appeared partially suppressible and became more evident during stress and voluntary movements. Cognitive examination revealed frontal-executive dysfunction, memory impairment, and attention deficit. Brain magnetic resonance imaging (MRI) disclosed signs of posterior periventricular chronic cerebrovascular disease and a marked ponto-cerebellar atrophy, as confirmed by volumetric MRI analysis. A dopamine transporter imaging scan demonstrated a bilaterally reduced putamen and caudate nucleus uptake. Ataxin-2 (*ATXN2*) gene analysis revealed a 36 cytosine-adenine-guanine (CAG) repeat expansion, confirming the diagnosis of SCA2.

**Conclusions:**

SCA2 should be considered among the possible causes of adult-onset oro-facial dyskinesia, especially when the family history suggests an inherited cerebellar disorder. Additional clinical features, including parkinsonism and motor neuron disease, may represent relevant cues for an early diagnosis and adequate management.

## Background

Spontaneous oro-facial dyskinesias have been reported in the elderly, with a prevalence rate ranging from 1.5 to 38.0% [1]. In particular, dyskinesias are known to occur in approximately 15–30% of patients receiving long-term treatment with neuroleptic drugs, which represent the main cause of oro-facial dyskinesia in adults.

Genetic familiar causes of oro-facial dyskinesia are usually restricted to Huntington’s disease, being the autosomal dominant pattern one of the diagnostic keys, whereas other causes are often missed or underestimated. Here, we describe a case of isolated late-onset oro-facial dyskinesia in an elderly patient with a genetic diagnosis of Spinocerebellar Ataxia type-2 (SCA2).

## Case presentation

A 75-year-old man was referred to our Neurology Department because of a progressive balance difficulty from the age of 60 years, associated with involuntary movements of the mouth and tongue over the last 3 months. These movements were mainly noted by his family members and did not cause significant distress to the patient’s activities of daily living. More recently, he also complained some swallowing disturbance and speech difficulty. His past medical history was negative and no exposure to anti-dopaminergic agents, other neuroleptic drugs, antidepressant medications (such as tricyclic antidepressants and selective serotonin reuptake inhibitors), or other drugs was present or reported. Family history was positive for SCA2; the patient provided information on two affected family members (both deceased at the time of the patient’s examination), i.e. a brother who developed a progressive walking impairment at the age of 56 years, and the son of whom who presented with gait disturbance from the age of 18 years. The genetic analysis performed in these relatives revealed a number of 39 and 49 cytosine-adenine-guanine (CAG) repeat expansions in the Ataxin-2 (*ATXN2)* gene, respectively.

Proband’s clinical examination showed saccadic pursuit, gait and trunk ataxia, limb dysmetria, dysarthria, mildly increased tone in all limbs, upper limb hyporeflexia and lower limb hyperreflexia; atrophy of the right first dorsal interosseous muscle and of the right supraspinatus muscle, together with fasciculations of the shoulders, were also evident. At rest, isolated involuntary masticatory movements of the jaw, mouth, and tongue were noted and appeared more evident during stress and voluntary movements. Additionally, they were very frequent, of short duration, partially suppressible, and not associated with any sensory component or dyskinetic/hyperkinetic and other dysfunctional movement of the face or other parts of the body.

**Fig. 1:**
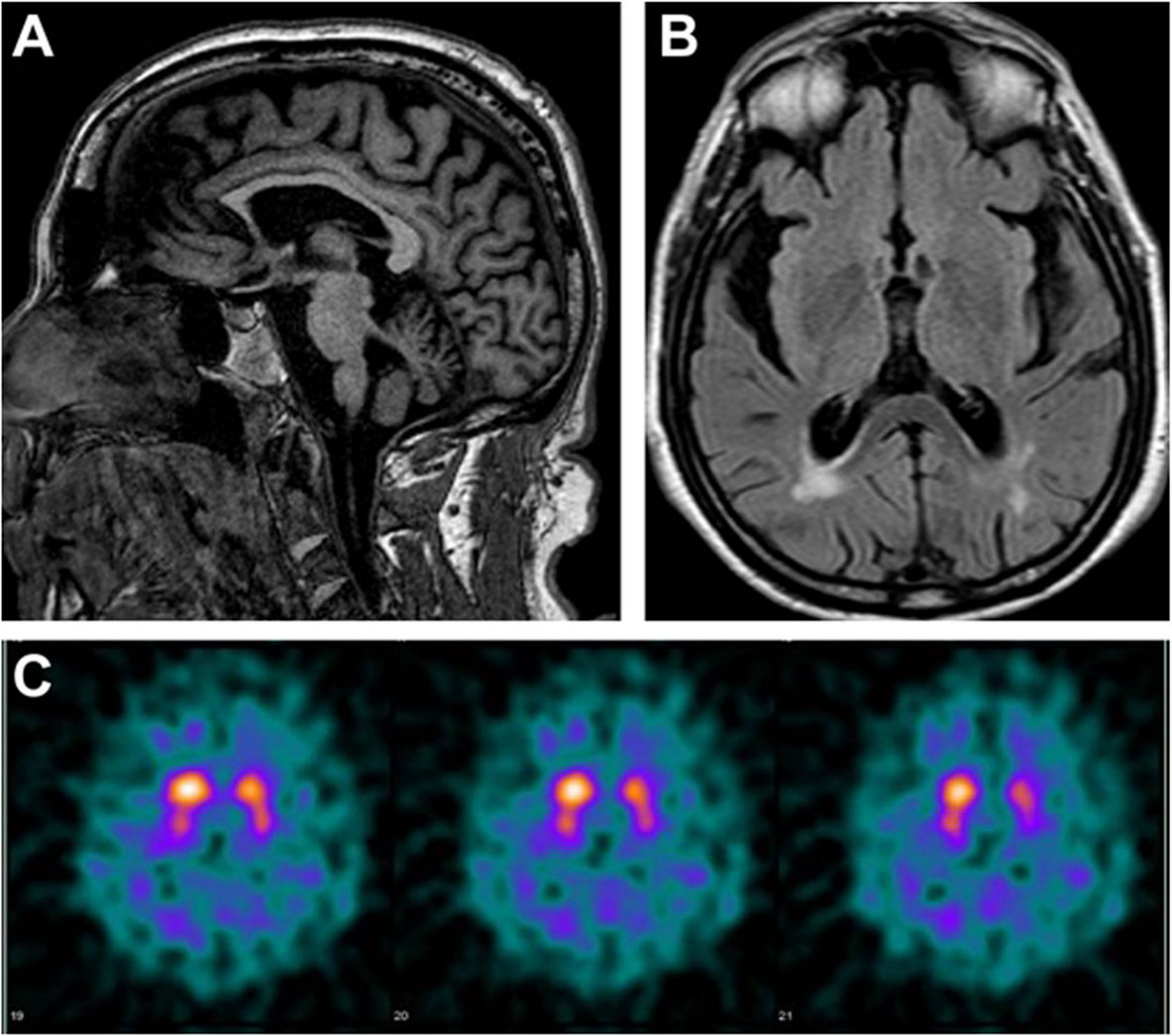
Brain magnetic resonance imaging showing: **a** marked ponto-cerebellar atrophy, and **b** signs of chronic cerebrovascular disease. Dopamine transporter imaging scan (**c**) showing bilaterally reduced putamen and caudate nucleus uptake

Cognitive examination revealed frontal-executive dysfunction, memory impairment, and attention deficit. Extensive blood tests were unremarkable, except for a mild increase in glycated hemoglobin. Cerebrospinal fluid analysis was normal. Electromyography showed bilateral fasciculations of the deltoid, brachialis, extensor digitorum communis, abductor pollicis brevis, first dorsal interosseous, vastus medialis, tibialis anterior, and medial gastrocnemius muscles. A prolonged central motor conduction time from the left tibialis anterior muscle was evident to transcranial magnetic stimulation. Visual evoked potentials showed increased latency of the P100 wave, bilaterally. Brainstem auditory evoked potentials were abnormal on the left side and somatosensory evoked potentials were compatible with a diffuse alteration of the central sensory pathway. Brain magnetic resonance imaging (MRI) disclosed posterior periventricular signs of chronic cerebrovascular disease and a marked ponto-cerebellar atrophy (Fig. 1a-b), as confirmed by volumetric MRI analysis. Because of the mentioned movement disorders, a dopamine transporter imaging scan was also performed (Fig. 1c) and demonstrated a bilaterally reduced uptake of the putamen and caudate nucleus. Based on patient’s family history, *ATXN2* gene analysis was carried out and revealed a number of 36 CAG repeat expansion, thus confirming the diagnosis of SCA2.

## Discussion and conclusion

The occurrence of movement disorders in SCA patients provides useful hints towards the possible underlying genotype. Although chorea and dystonia have been reported in 38% of cases of SCA2 [2], the association with isolated oro-facial dyskinesia has not been previously described. To our knowledge, the occurrence of oro-facial dyskinesia in SCA2 was reported in a 43-year-old woman only, who presented with a long history of progressive ataxia [3]. However, unlike the patient described here, she exhibited a complex movement disorder, also involving upper limbs and head tremor, and associated with the lack of teeth. The authors hypothesized that the long disease progression, ataxia severity, and edentulism might have contributed to this phenomenology [3].

As known, the cerebellar degeneration can be responsible for some movement disorders observed in SCA patients, such as tremor, dystonia, and myoclonus, whereas in other cases these clinical manifestations are more likely due to a neuronal loss within the substantia nigra, pallidum, caudate nucleus, motor cortex, and thalamus [4, 5]. In the case reported here, the occurrence of oro-facial dyskinesia might be related to a degeneration of the putamina and caudate nuclei, as suggested by the finding of abnormal dopamine transporter imaging.

Upper and lower motor neuron involvement was also noted in this patient. It is known that an intermediate length of *ATXN2* CAG repeats is associated with sporadic Amyotrophic Lateral Sclerosis (ALS), supporting the existence of an overlapping clinical picture between ALS and SCA2. Indeed, several studies suggested an increased risk for ALS when alleles have 31, 32, or 33 repeats; for alleles with 34 or more repeats, as in the present case, some individuals may present with motor neuron disease in addition to the cerebellar features [6]. Moreover, a number of 36 repeats in the *ATXN2* gene is usually associated with parkinsonian features, as observed in our patient and confirmed by functional neuroimaging studies [7].

It is worth noting that the symptom description might resemble a psychogenic tic as well. However, although dyskinesia may start as mild shakes, tremors, or even tics, these usually occur in younger people and with a variety of intermittent, sudden, or repetitive stereotyped movements, often triggered by external stimuli. Moreover, although tics can become automatic behaviors over time, the individual can initially control and even reduce them [8]. Finally, unlike the present case, adult-onset tic disorders are associated with grinding/clenching of the teeth or lack of teeth [9].

A caveat of this report is that dyskinesia was restricted to the oro-mandibular area, thus making it difficult to rule out a persistent dyskinesia secondary to a drug exposure. However, since this type of dyskinesia can be persistent even after the discontinuation of the causative drugs, special attention was paid to the patient’s drug history, who excluded the exposure to any potential dyskinesia-inducing substance, as confirmed also by the caregiver and the past medical records.

In conclusion, we suggest that SCA2 should be considered among the possible causes of adult-onset oro-facial dyskinesia, especially when the family history supports an inherited cerebellar disorder. Additional clinical features, including parkinsonism and motor neuron disease, may represent relevant cues for early diagnosis and adequate management of these patients.

## Data Availability

All data generated or analyzed during this study are included in this published article.

## Abbreviations

ALS: Amyotrophic Lateral Sclerosis
*ATXN2*: Ataxin-2
CAG: cytosine-adenine-guanine
MRI: magnetic resonance imaging
SCA2: Spinocerebellar Ataxia type 2
